# Mapping Short Association Fibers in the Early Cortical Visual Processing Stream Using In Vivo Diffusion Tractography

**DOI:** 10.1093/cercor/bhaa049

**Published:** 2020-04-08

**Authors:** Fakhereh Movahedian Attar, Evgeniya Kirilina, Daniel Haenelt, Kerrin J Pine, Robert Trampel, Luke J Edwards, Nikolaus Weiskopf

**Affiliations:** 1 Department of Neurophysics, Max Planck Institute for Human Cognitive and Brain Sciences, 04103 Leipzig, Germany; 2 Department of Education and Psychology, Center for Cognitive Neuroscience Berlin, Free University Berlin, 14195 Berlin, Germany; 3 Felix Bloch Institute for Solid State Physics, Faculty of Physics and Earth Sciences, Leipzig University, 04109 Leipzig, Germany

**Keywords:** retinotopy, subcortical, submillimeter resolution, superficial white matter, U-fibers

## Abstract

Short association fibers (U-fibers) connect proximal cortical areas and constitute the majority of white matter connections in the human brain. U-fibers play an important role in brain development, function, and pathology but are underrepresented in current descriptions of the human brain connectome, primarily due to methodological challenges in diffusion magnetic resonance imaging (dMRI) of these fibers. High spatial resolution and dedicated fiber and tractography models are required to reliably map the U-fibers. Moreover, limited quantitative knowledge of their geometry and distribution makes validation of U-fiber tractography challenging. Submillimeter resolution diffusion MRI—facilitated by a cutting-edge MRI scanner with 300 mT/m maximum gradient amplitude—was used to map U-fiber connectivity between primary and secondary visual cortical areas (V1 and V2, respectively) in vivo. V1 and V2 retinotopic maps were obtained using functional MRI at 7T. The mapped V1–V2 connectivity was retinotopically organized, demonstrating higher connectivity for retinotopically corresponding areas in V1 and V2 as expected. The results were highly reproducible, as demonstrated by repeated measurements in the same participants and by an independent replication group study. This study demonstrates a robust U-fiber connectivity mapping in vivo and is an important step toward construction of a more complete human brain connectome.

## Introduction

Short association fibers (U-fibers) are cortico-cortical white matter fibers connecting primarily adjacent cortical areas (Meynert 1885) with estimated lengths of 3–30 mm (Schüz and Braitenberg 2002). These fibers are located directly below the cortical gray matter–white matter (GM–WM) boundary in the superficial white matter (SWM) and follow the pattern of cortical folding; they are hence also called U-fibers. The estimated contribution of U-fibers to the total number of all white matter fibers in the human brain is almost 90% (Schüz and Braitenberg 2002). These fibers play an important role in brain development and aging (Phillips et al. 2013; Wu et al. 2014, 2016; Nazeri et al. 2015), function (e.g., sensory–motor integration [Catani et al. 2011, 2017] and language processing [Friederici 2011]), and pathology including Alzheimer’s disease (Fornari et al. 2012; Carmeli et al. 2014; Reginold et al. 2016; Phillips et al. 2016a), temporal lobe epilepsy (Liu et al. 2016), Huntington’s disease (Phillips et al. 2016b), autism spectrum disorder (Wilkinson et al. 2016), and nonlesional epilepsy (O’Halloran et al. 2017).

Despite their significance, U-fibers are highly underrepresented in current descriptions of the human brain connectome owing to the challenges in mapping them using available postmortem and in vivo methods. These fibers have been identified in the postmortem brain in the frontal (Jakob 1906; Catani et al. 2011), parietal (Catani et al. 2017), and occipital (Sachs 1892; Vergani et al. 2014) lobes using Klingler’s dissection (Klingler 1935) and histology. However, the qualitative and/or 2D nature of these techniques did not allow for comprehensive connectivity mapping. U-fiber mapping in vivo has been limited by methodological challenges in diffusion-weighted imaging (DWI) of these fibers (Song et al. 2014) and in their validation.

Diffusion MRI tractography (Conturo et al. 1999; Basser et al. 2000; Bammer et al. 2003; Beaulieu 2006) is currently the only technique for mapping human structural brain connectivity in vivo and may in principle be used to map U-fiber connectivity as demonstrated previously using conventional diffusion MRI tractography (Conturo et al. 1999; Catani et al. 2002, 2011, 2017; Lawes et al. 2008; Oishi et al. 2008; Zhang et al. 2010; Guevara et al. 2011, 2012; Magro et al. 2012; Pron et al. 2018). The validation of in vivo U-fiber connectivity mapping is challenging because tractography is generally difficult to validate (Fillard et al. 2011; Johansen-Berg and Behrens 2011; Côté et al. 2013; Hubbard and Parker 2013; Knösche et al. 2015; Maier-Hein et al. 2017; Sotiropoulos and Andrew 2017; Jeurissen et al. 2019; Schilling et al. 2019) and quantitative knowledge of U-fiber geometry and distribution is limited.

Submillimeter resolution DWI is required to comprehensively map the short association fibers (Song et al. 2014) because of their short lengths and location at the GM–WM boundary in superficial white matter (SWM) where partial volume effects (Alexander et al. 2001; Vos et al. 2011) and complex fiber crossings (Jeurissen et al. 2013; Reveley et al. 2015) should be addressed. The spatial resolution of conventional diffusion MRI is limited by the available signal-to-noise ratio (SNR), which is low due to the long echo times (TE) caused by the need for long diffusion weighting gradients. However, the recent development of high-performance gradients (gradient amplitudes up to 300 mT/m) in whole-body human MRI scanners (Setsompop et al. 2013) allows for a significant reduction of TE (Jones et al. 2018). The consequently higher SNR facilitates acquisition of submillimeter resolution in vivo diffusion MRI, potentially enabling robust U-fiber mapping.

Qualitative postmortem dissections have been used previously to validate in vivo U-fiber connectivity maps for selected short association fibers (Catani et al. 2011) and to qualitatively reproduce early histologically derived maps of short connections in the occipital lobe (Vergani et al. 2014). Validation based on postmortem techniques is qualitative, is prone to tissue changes (Edwards et al. 2018), and, outside of animal models, cannot be systematically performed in the same individual. We propose an alternative important step toward validation that can be performed by mapping the short association fibers in areas where anatomical and functional connections are well characterized.

It is known that in the primate brain, the flow of visual information between primary and secondary visual cortical areas (V1 and V2, respectively) follows the principle of retinotopic projection (Holmes 1918, 1945; Tootell et al. 1988; Felleman and Van Essen 1991; Sereno et al. 1995; DeYoe et al. 1996; Engel et al. 1997; Wandell et al. 2007; Wandell and Winawer 2011). The principle of retinotopic projection suggests highly efficient connectivity via short fibers between retinotopically corresponding areas in V1 and V2 and has been demonstrated in the macaque brain using tracer injection (Van Essen et al. 1982). Tracer-injection studies in the macaque visual cortex revealed strong axonal connections within V1, within V2, and between V1 and higher visual cortical areas V2, V3, as well as MT (Felleman and Van Essen 1991; Levitt et al. 1994; Coogan and Van Essen 1996; Angelucci et al. 2002; Stettler et al. 2002). In macaque, lateral connectivity, that is, connectivity between neighboring receptive fields, within V1 is facilitated by intracortical fibers up to 7 mm in range end to end (Stettler et al. 2002). Quantitative tracer-injection studies have demonstrated that the majority of the neurons in V1 project to V2 in the macaque brain (Barone et al. 2000) and that the detected feedback connections from V2 to V1 are also retinotopically organized but with larger receptive areas spanning up to 6 mm in range within the cortex (Stettler et al. 2002).

The detailed map of the connections in the macaque visual system (Felleman and Van Essen 1991; Van Essen et al. 1992) has been used for validation of ex vivo DWI tractography in macaque (Azadbakht et al. 2015). Moreover, the retinotopic organization of the optic radiation tract projecting into V1 was used for validation of a tractography method (Aydogan and Shi 2018). Therefore, these well-known connectivity patterns in the early cortical visual processing stream, comprising V1 and V2, can be considered a suitable test bed for in vivo U-fiber connectivity mapping using diffusion MRI tractography.

Functional MRI has been used in conjunction with in vivo diffusion MRI tractography to study white matter connectivity and organization in relation to visual field maps in the primate visual system (see review by Rokem et al. 2017). The connectivity between the intraparietal sulcus and early visual cortical areas V1, V2, and V3 was demonstrated in the human brain and showed a retinotopic tendency, especially for connectivity to V1 (Greenberg et al. 2012). Organization of occipital-callosal fibers connecting the left and right hemispheric occipital cortices was also demonstrated and revealed a dorsal (via V3 and V7) to ventral (via V4) arrangement as confirmed by tractography clustering using visual field maps (Dougherty et al. 2005). The vertical occipital fasciculus (VOF)—the major white matter fiber pathway connecting the dorsal (V3) and ventral (V4) visual cortices—was mapped, and its endpoints were localized using visual field maps (Yeatman et al. 2014; Takemura et al. 2015). However, these studies focused on mapping only the long-range connections. The short-range V1–V2 connectivity mediated by white matter association fibers has not yet been explored with in vivo DWI tractography.

In this study, we present an in vivo U-fiber connectivity mapping approach and an important step toward validation by combining submillimeter resolution diffusion-weighted imaging (DWI) probabilistic tractography with a priori anatomical knowledge and functional MRI retinotopy (Sereno et al. 1995; Engel et al. 1997) in the human early cortical visual processing stream. In particular, we expect short association fibers (estimated by relative streamline counts) to preferentially connect retinotopically corresponding areas in V1 and V2. This integrated in vivo approach exploits the power of functional MRI to robustly map the retinotopic organization of V1 and V2 and leverages known cortico-cortical connectivity patterns—here, retinotopically organized connectivity in the human early cortical visual processing stream—to validate U-fiber connectivity obtained with diffusion MRI. This study further demonstrates the feasibility and reproducibility of our multimodality approach for accurate and robust in vivo U-fiber connectivity mapping.

## Materials and Methods

### Subject Information

Two independent experiments were performed on two different groups of healthy participants with no reported history of neurological disease. The participants gave written informed consent before participation in the study, which was approved by the ethics committee of the Medical Faculty at Leipzig University.

In “Experiment 1,” short association fibers connecting V1 and V2 cortical areas were mapped in a small group of three participants (two females, one male; 27 ± 2 years) as a proof of concept. For each participant, three diffusion MRI (dMRI) acquisitions—two acquired with a 32-channel radio-frequency (RF) receive head coil and one acquired with a flexible surface 23-channel RF receive coil (Frass-Kriegl et al. 2018)—on a 3-T Connectom scanner and one functional MRI (fMRI) acquisition on a 7-T scanner were performed. Each scan was performed on a different day.

In “Experiment 2,” reproducibility and robustness of findings in the small group were assessed in a larger group of 14 participants (seven females, seven males; 26 ± 3 years). For each participant in the second experiment, two dMRI acquisitions—one acquired with the 32-channel RF receive head coil and the other with the flexible surface RF receive coil—and one fMRI acquisition were performed. The fMRI was originally acquired for a different study; the stimulus setup and acquisition parameters were therefore slightly different to those in Experiment 1, though with no effect on the quality of retinotopy. Each scan was performed on a different day. For each participant in both experiments, the time elapsed between the dMRI scans was on average 1.5 weeks (maximally 1.5 months) and that between the dMRI and fMRI scans was on average 3 months (maximally 6.5 months).

### Functional MRI

#### Visual Stimulation Paradigm

Stimuli were presented onto a front-projection screen positioned above the participant’s chest and viewed through a mirror mounted on the 32-channel RF receive head coil.

In Experiment 1, a phase-encoded paradigm (Sereno et al. 1995; Engel et al. 1997) was used with a contrast-reversing (4 Hz), randomly colored, checkerboard stimulus that was restricted to a clockwise/anticlockwise rotating ray (width: 30°) or an expanding/contracting ring presented in separated runs. In each run, 8.25 rotation cycles were presented on the screen with a cycle period of 36 and 60 s for the moving ring (eccentricity mapping) and the rotating ray (polar angle mapping), respectively. Participants were asked to maintain their gaze at a central fixation point throughout all runs. To maintain peripheral attention during central fixation, numbers and letters were randomly presented on the screen within the rings or rays. Participants were asked to press a button when seeing a number (Sereno et al. 2013).

In Experiment 2, a phase-encoded paradigm (Sereno et al. 1995; Engel et al. 1997) was used with a black-and-white checkerboard stimulus that was restricted to a clockwise/anticlockwise rotating ray (width: 30°) or an expanding/contracting ring presented in separated runs. In each run, 8.25 rotation cycles were presented on the screen with a cycle period of 32 and 64 s for the moving ring (eccentricity mapping) and the rotating ray (polar angle mapping), respectively. Participants were asked to maintain their gaze at a central fixation point throughout all runs. No task was performed by the participants.

#### 7-T fMRI Acquisition

fMRI was acquired on a 7-T Magnetom (Siemens Healthineers) whole-body scanner using a single-channel-transmit/32-channel RF receive head coil (Nova Medical). fMRI data were recorded with an isotropic spatial resolution of 1 mm with a single-shot gradient-echo echo-planar imaging (GE-EPI) sequence using the following imaging parameters for Experiment 1: excitation flip angle = 78°, echo time (TE) = 26 ms, repetition time (TR) = 3000 ms, partial Fourier = 7/8, in-plane acceleration factor = 4 with generalized autocalibrating partially parallel acquisition (GRAPPA) reconstruction (Griswold et al. 2002), readout bandwidth = 1184 Hz/Px, echo spacing = 1 ms, acquisition matrix = 192 × 192, 56 coronal oblique slices, distance factor = 0%, and inferior–superior phase encoding (PE) direction. The slice slab was positioned to cover the visual areas V1, V2, and V3 and the posterior parts of ventral and dorsal V4 in the occipital lobe. For Experiment 2, the following imaging parameters were used: excitation flip angle = 68°, echo time (TE) = 21 ms, repetition time (TR) = 2000 ms, partial Fourier = 6/8, in-plane acceleration factor = 3 with GRAPPA, readout bandwidth = 1164 Hz/Px, echo spacing = 1 ms, acquisition matrix = 148 × 148, 40 coronal slices, distance factor = 0%, and inferior–superior phase encoding (PE) direction. The slice slab was positioned to cover the visual areas V1 and V2. A structural image was acquired for cortical surface reconstruction and registration between diffusion and functional MRI data using a magnetization-prepared rapid gradient-echo (MP2RAGE) sequence with sagittal orientation (Marques et al. 2010): voxel size = 0.7 × 0.7 × 0.7 mm^3^, TE = 2.45 ms, TR = 5000 ms, partial Fourier = 6/8, in-plane acceleration factor = 2 with GRAPPA reconstruction in anterior–posterior (AP) primary PE direction, readout bandwidth = 250 Hz/Px, echo spacing = 6.8 ms, acquisition matrix = 224 × 224, 240 slices, distance factor = 50%, and fat suppression enabled. Nonselective adiabatic inversion pulses were applied before rapid gradient-echo measurements at the inversion times TI1/2 = 900 ms/2750 ms with excitation flip angles = 5° and 3°, respectively. The total acquisition time was approximately 28 min for fMRI and approximately 11 min for MP2RAGE imaging.

#### fMRI Analysis

Analysis of fMRI data was performed using SPM12 (Functional Imaging Laboratory, University College London; Penny et al. 2006), FreeSurfer 6.0 (Fischl 2012), and ANTs 2.2.0 (Avants et al. 2009) software packages and the gradunwarp toolbox (https://github.com/Washington-University/gradunwarp) (Glasser et al. 2013), as described below.

#### Preprocessing

The functional time series were corrected for within- and between-run motion using SPM12. The time series were then baseline corrected using a high-pass filter implemented in SPM12 with 1/120 and 1/72 Hz cutoff frequencies in Experiment 1 and 1/192 and 1/96 Hz cutoff frequencies in Experiment 2 for polar angle and eccentricity mapping runs, respectively.

#### Retinotopic Mapping

**Figure 1:**
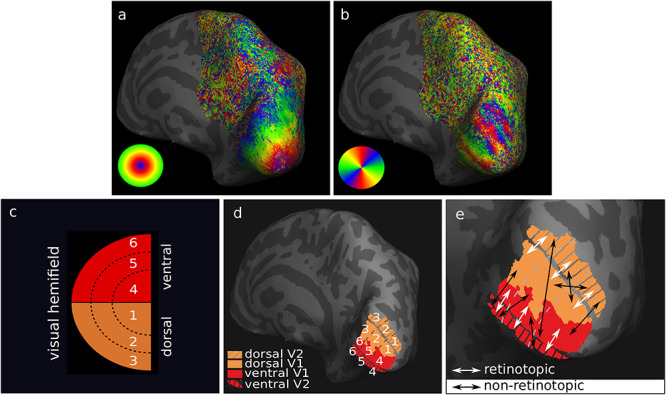
Retinotopic segmentation of V1 and V2 cortical borders was enabled by functional MRI retinotopy. Images are shown on the inflated surface of the right hemisphere for one participant and cortical curvature is shown with dark (sulci) and light (gyri) gray. (*a*) Eccentricity and (*b*) polar angle maps were obtained, and (*c*) schematic projections of the six subdivisions of V1 and V2 visual hemifields were represented by the corresponding (*d*) six retinotopically segmented V1 and V2 areas. (*e*) Connectivity between the six V1 and V2 segments was defined as retinotopic (white arrows) for retinotopically corresponding connectivity and non-retinotopic (black arrows) for non-retinotopic connectivity on the inflated cortical surface.

The V1 and V2 cortical areas were mapped to cortical surfaces as follows. The *T*_1_-weighted (*T*_1_w) structural image from the MP2RAGE was used for cortical segmentation and surface reconstruction. The image was first corrected for gradient nonlinearity distortions using the gradunwarp toolbox (Glasser et al. 2013). Background noise was then removed using a regularization approach (O'Brien et al. 2014), and the bias field was corrected using SPM12. Segmentation of each hemisphere was performed using FreeSurfer (Dale et al. 1999) at the original image resolution, and the midcortical surface at 50% cortical depth was computed (Fischl et al. 1999). The transform between MP2RAGE and GE-EPI functional MRI space was computed using ANTs in two steps. First, a rigid-body (six-parameter) transform was estimated, which was then used to initialize estimation of the nonlinear transform. The inverse nonlinear transform was then applied to the eccentricity and the polar angle phase maps obtained from the retinotopy experiment (Fig. 1*a*,*b*) using linear interpolation, and the transformed volumes were sampled onto the midcortical surface at 50% cortical depth using nearest-neighbor interpolation. Registration was restricted to rigid body only where nonlinear transformation failed, as assessed visually.

The V1 and V2 cortical borders were defined on visual field sign maps (Sereno et al. 1995) using the eccentricity and polar angle phase maps. The first quarter of the stimulus cycle was discarded from each time series and excluded from further analysis. Then, each voxel’s time series was Fourier transformed, and runs with opposite stimulus movement direction were averaged to compensate for the hemodynamic delay. Finally, for each location on the cortical surface, the phase lag of the stimulus response in eccentricity and polar angle directions was computed from the averaged Fourier component at the stimulus frequency, enabling the computation of visual field sign maps (Sereno et al. 1995) on which the functional definition of V1 and V2 borders was defined.

Six retinotopic segments of V1 and V2 corresponding to six subdivisions of the visual hemifield (Fig. 1*c*) were created as follows. The visual field sign map was computed using the sampled retinotopy data (Sereno et al. 1995) (Supplementary Fig. 2*a*). Based on the visual field sign map, masks including the stimulated portion of V1 and V2 were manually defined (Supplementary Fig. 2*b*). Six retinotopic segments of V1 and V2 were then defined using the eccentricity and polar angle information. This was achieved in Experiment 1 by first scaling the V1 and V2 phase maps to the interval (0, 1) followed by thresholding the polar angle data into two and the eccentricity data into three equal parts, respectively (Fig. 1*d*; Supplementary Fig. 2*c*). The borders of the six V1 and V2 retinotopic segments were manually refined. In Experiment 2, the eccentricity and polar angle maps were used directly to manually define the six retinotopic segments by following the polar angle and isoeccentricity lines. The manual approach—which in principle followed the same concept used in Experiment 1—had to be employed because accurate scaling of the V1 and V2 phase maps could not be achieved for all participants in Experiment 2, potentially due to noisy regions in the phase maps that rendered the scaling unstable. Results of retinotopic mapping in Experiments 1 and 2 were visually compared and proved consistent. Then, the final V1 and V2 retinotopic segments corresponding to the six subdivisions of the visual hemifield were transformed into the volumetric space of the segmented anatomy.

#### Mapping to Diffusion MRI Space

The transform between MP2RAGE and diffusion MRI space was computed by rigid (6 parameters) registration in Experiment 1 and rigid (6 parameters) followed by affine (12 parameters) and then nonlinear registration in a single interpolation step in Experiment 2 between the MP2RAGE *T*_1_w image and the diffusion MRI-derived fractional anisotropy (FA) map using ANTs. Multistep registration was used in Experiment 2 to obtain high-quality registration for all subjects in the larger group. The computed transformation was used to register the V1 and V2 retinotopic segments to the diffusion MRI space for subsequent structural connectivity mapping between V1 and V2 (Supplementary Fig. 2*d–f*). V1–V2 connectivity was defined as retinotopic for corresponding V1 and V2 retinotopic segments and non-retinotopic for noncorresponding V1 and V2 retinotopic segments (Fig. 1*e*).

### Diffusion MRI

#### 3-T Connectom dMRI Acquisition

DWI was acquired on a 3-T Connectom (Siemens Healthineers) scanner with 300 mT/m maximum gradient amplitude using either a 32-channel whole-brain RF receive head coil (referred to as the 32-channel coil hereafter) or a flexible surface 23-channel RF receive coil (referred to as the flexible surface coil hereafter) tailored for high-sensitivity imaging of the occipital cortex (Frass-Kriegl et al. 2018; Kirilina et al. 2018). The flexible surface coil was placed tightly around the back of the participants’ heads using thin padding in order to obtain high SNR proximal to the coil surface in superficial brain areas (Supplementary Fig. 1*a*).

In Experiment 1, DWI was acquired in three sessions, each performed on a different day for each participant. The 32-channel coil was used for two sessions (for scan–rescan), and the flexible surface coil was used for the third session. In Experiment 2, DWI was acquired in two sessions, each performed on a different day for each participant. The 32-channel coil was used for one session and the flexible surface coil was used for the other.

DWI was acquired with 0.8-mm isotropic resolution to enhance U-fiber detectability (Song et al. 2014) for both RF receive coils. Two interleaved diffusion weighting shells (*b* = 800 s/mm^2^ and *b* = 1800 s/mm^2^) were acquired to enable partial volume effect estimation and crossing fiber modeling. DWI was acquired along 60 noncolinear diffusion encoding directions (Jones et al. 1999) for each shell using a Stejskal–Tanner diffusion encoding scheme. The diffusion encoding directions were distributed uniformly over the whole sphere for optimal correction of “eddy” current (EC)-induced geometric distortions (Andersson and Sotiropoulos 2016). A total of 30 nondiffusion-weighted images (*b* = 0 s/mm^2^) were recorded, 25 of which were interspersed with the acquisition of the DWI (after every 10 DWI images). The DWI acquisition was repeated twice per session to improve the SNR. DWI was acquired with an anterior–posterior (AP) PE direction. For correction of susceptibility-related distortions, an additional *b* = 0 s/mm^2^ image was acquired in Experiment 1 between the two AP acquisitions and in Experiment 2 prior to the two AP acquisitions with reversed PE in the posterior–anterior (PA) direction (Andersson et al. 2003; Smith et al. 2004). The total DWI acquisition time was approximately 45 min per session. DWI acquisition was optimized for SNR by minimizing the echo time (TE) via enabling partial Fourier factor of 5/8, parallel imaging in the PE direction with acceleration factor 2 for imaging with the 32-channel coil, and using restricted field of view (FOV) in the PE direction for imaging with the flexible surface coil.

For DWI acquired using the 32-channel coil, a single-shot 2D spin-echo echo-planar imaging (SE-EPI) sequence was used (Harvard Medical School, Massachusetts General Hospital [MGH] [Setsompop et al. 2012a, 2013]). The following imaging parameters were used: excitation flip angle = 90°, TE = 66 ms, TR = 8900 ms, partial Fourier factor in PE direction = 5/8, in-plane acceleration factor 2 in PE direction using GRAPPA reconstruction, readout bandwidth (BW) = 1148 Hz/Px, echo spacing = 0.96 ms, acquisition matrix = 256 × 256, field of view (FOV) = 206 × 206 mm, and fat saturation enabled. A slab containing 62 oblique near-axial slices was placed to cover the V1 and V2 cortical areas.

For DWI acquired using the flexible surface coil, a single-shot limited FOV 2D SE-EPI sequence was used (Center for Magnetic Resonance Research [CMRR], University of Minnesota, Development Release R016a [https://www.cmrr.um n.edu/multiband/] [Moeller et al. 2010; Feinberg et al. 2010]). The low sensitivity of the flexible surface coil in frontal brain areas, combined with an RF saturation pulse covering the frontal part of the brain applied prior to the acquisition of each slice, allowed us to limit the FOV to 85 mm in the PE direction without fold-over artifacts. The following imaging parameters (closely matching the parameters for the 32-channel coil acquisitions) were used: excitation flip angle = 90°, TE = 65.2 ms, TR = 8710 ms, partial Fourier factor in PE direction = 5/8, no in-plane acceleration, readout BW = 1220 Hz/Px, echo spacing = 0.98 ms, acquisition matrix = 256 × 106, FOV = 206 × 85 mm, and fat saturation enabled. A slab containing 64 oblique near-axial slices was placed to cover the V1 and V2 cortical areas.

#### dMRI Analysis

Analysis of diffusion MRI data was performed using MATLAB version R2017b, MRtrix 3.0 (Tournier et al. 2019) (http://www.mrtrix.org/), FSL 5.0.9 (Smith et al. 2004; Woolrich et al. 2009; Jenkinson et al. 2012), and ANTs 2.2.0 (Avants et al. 2009) software packages and the gradunwarp toolbox (https://github.com/Washington-University/gradunwarp) (Sotiropoulos et al. 2013), as described below.

#### Quality Control

Images were visually inspected for artifacts arising from displaced fat signal—the flexible surface coil is particularly sensitive to the fat in the scalp near the coil surface—in addition to severe motion and other sources, during acquisition. To address the displaced fat signal artifact, the imaging slab was oriented in a way to shift the artifact away from the regions of interest (ROIs) in experiments using the flexible surface coil. DWI acquired with the flexible surface coil was excluded from the study if the displaced fat signal was present in the regions of interest. All other DWI were considered valid. As an additional quantitative data quality assessment, the time-series *b* = 0 s/mm^2^ images were used to estimate the temporal SNR (tSNR). For each coil, the voxel-wise tSNR was computed as the ratio of the temporal signal mean and standard deviation. Two regions of interest (ROIs) were defined in corresponding anatomical slices in the tSNR maps, one proximal and the other distal to the coil surface, that is, one ca. 2–3 cm and the other more than 5 cm away from the coil surface, respectively (Supplementary Fig. 1*b*). Aggregate tSNR statistics (mean ± standard deviation [SD]) across voxels were reported for each ROI (Supplementary Fig. 1*c*).

Based on Experiment 2 with a larger group, we examined whether the extent of motion estimated for the 32-channel and flexible surface coil DWI was different because of differences in head fixation. Motion parameters were estimated using the FSL eddy algorithm and compared between the 32-channel and flexible surface coil DWI using a paired group *t*-test performed on the standard deviations of each motion parameter computed across all DWI acquisitions for each participant.

#### Distortion Correction

The DWI was denoised (Veraart et al. 2016b, 2016c) and corrected for Gibbs ringing (Perrone et al. 2015; Veraart et al. 2016a) artifacts using “dwidenoise” and “mrdegibbs” functions implemented in MRtrix. The DWI was then simultaneously corrected for bulk head motion, eddy current (EC), and tissue susceptibility–induced off-resonance geometric distortions with outlier replacement (Andersson et al. 2003, 2016; Andersson and Sotiropoulos 2016) using the FSL eddy_cuda algorithm, with rotation of the B-matrix (Leemans and Jones 2009). The off-resonance field created by tissue susceptibility variations was estimated using the reversed PE *b* = 0 s/mm^2^ image pair with FSL top-up (Andersson et al. 2003; Smith et al. 2004) and used as input to eddy_cuda. A quadratic model of the EC field was assumed. The DWI was corrected for gradient nonlinearity-induced geometric distortions using the gradunwarp toolbox and the 3-T Connectom gradient field specifications provided by the manufacturer (Sotiropoulos et al. 2013). The DWI bias field created by the nonuniform coil receive sensitivity was corrected for using the “dwibiascorrect” function implemented in MRtrix based on the “N4” algorithm implemented in ANTs (Tustison et al. 2010). For the flexible surface coil scans, the bias field was estimated from regions with tSNR greater than 3 in *b* = 0 s/mm^2^ images only (see Supplementary Fig. 1*b*).

#### Fiber Distribution Estimation

Fiber orientation distribution functions (fODFs) were estimated using the unsupervised multishell and multitissue constrained spherical deconvolution (MSMT-CSD) algorithm (Tournier et al. 2004, 2007; Jeurissen et al. 2014; Dhollander et al. 2016) implemented in MRtrix using *b* = 0, 800 and 1800 s/mm^2^ images and harmonic fits up to the eighth order. This model was used to account for partial volume effects (Alexander et al. 2001; Vos et al. 2011) and crossing fibers (Jeurissen et al. 2013) at the GM–WM interface. A previous study of V1–V2 connectivity in the macaque brain demonstrated improved tractography performance using MSMT-CSD tractography owing to modeling of the partial volume effects (Theaud et al. 2018).

#### Fiber Tractography

Fiber pathways were reconstructed using second-order integration probabilistic streamline tractography (Behrens et al. 2007; Tournier et al. 2012) implemented in MRtrix, designed to avoid overshoots in areas of high fiber curvature, for example, U-fibers (Tournier et al. 2010). Whole-brain (cropped to the limited FOV of flexible surface coil DWI) tractography was performed using regular seed locations within each voxel throughout the brain using a 2 × 2 × 2 seed resolution. Streamline tracking and termination criteria were defined using an angle threshold of 45° for the maximal curvature and an fODF peak threshold of 0.1 to exclude small fODF peaks potentially corresponding to noisy fiber estimates. The streamline lengths were restricted to 3–100 mm and tractography step size was set to 0.2 mm.

For qualitative assessment of the DWI quality, the optic radiation tract was delineated based on termination in the retinotopically defined V1 segments and a second user-defined ROI positioned in an approximate anatomical location in the WM corresponding to the optic radiation tract. The vertical occipital fasciculus (Yeatman et al. 2014) and the short fibers connecting the upper and lower banks of the calcarine sulcus were also manually delineated by user-defined ROIs created based on a previous work (Sachs 1892). Short U-shaped fiber tracks were also specifically targeted by using maximum streamline length and curvature thresholds of 25 cm and 90°, respectively, using an in-house MATLAB script.

### V1–V2 Connectivity

#### Connectivity Mapping

V1–V2 connectivity was mapped separately for each hemisphere and for each acquisition. For each hemisphere, V1–V2 connectivity was mapped between all pairs of the six retinotopically defined V1 and V2 segments (Fig. 1*d*; Supplementary Fig. 2*c*). A corresponding six-by-six connectivity matrix was created based on the streamline counts for each hemisphere as follows. Whole-brain tractograms were filtered using all pairs of volumetric V1 and V2 retinotopic segments transformed to the DWI space. Streamlines were only retained if they terminated in both V1 and V2 for each pair of segments and were discarded otherwise. Tractography streamlines were also discarded if they traversed the cerebrospinal fluid (CSF), as defined by a CSF mask derived from MP2RAGE segmentation. Connectivity “strength” was expressed as relative streamline counts and was computed for each pair of V1 and V2 retinotopic segments by normalizing the streamline counts to the total counts between all V1 and V2 pairs in that hemisphere. The final connectivity was reported as a percentage relative connectivity strength in the connectivity matrix for each hemisphere and acquisition.

#### Statistical Analysis

The retinotopic connectivity principle was assessed, hypothesizing preferential connectivity between retinotopically corresponding areas in V1 and V2 and assuming no hemispheric differences in V1–V2 connectivity patterns. In Experiment 1, a group-average connectivity matrix was created by averaging across the individual connectivity matrices obtained for each hemisphere for all DWI acquisitions. In Experiment 2, two group-average connectivity matrices were created by separately averaging across the individual connectivity matrices obtained for each hemisphere for 32-channel and flexible surface coil DWI acquisitions. The total group-average retinotopic and non-retinotopic connectivity (Fig. 1*e*) strengths were reported based on the group-average percentage connectivity matrix in each case.

The mean reciprocal length of all streamlines connecting each pair of V1 and V2 retinotopic segments was calculated for each hemisphere to investigate (as a first-order approximation) the potential bias of streamline tractography in our study toward the detection of short streamlines (Jones 2010; Jbabdi and Johansen-Berg 2011; Ercsey-Ravasz et al. 2013; Donahue et al. 2016; Schilling et al. 2018; Jeurissen et al. 2019). In Experiment 1, a corresponding group-average reciprocal length matrix was created by averaging across the reciprocal length matrices obtained for each hemisphere. In Experiment 2, corresponding group-average reciprocal length matrices were created by separately averaging across the reciprocal length matrices obtained for each hemisphere for 32-channel and flexible surface coil scans.

For the small group scanned in Experiment 1, the statistical significance of the detected V1–V2 connectivity patterns was assessed by performing permutation *t*-tests as follows. First, the total retinotopic and total non-retinotopic connectivity strength was estimated for each hemisphere in each session by averaging across all retinotopic and non-retinotopic connectivity strengths within each connectivity matrix, respectively. A one-tailed *t*-test was then carried out over the six hemispheres (=two hemispheres per participant × three participants) based on the hypothesis that retinotopic connectivity was higher than non-retinotopic connectivity, yielding a *t* value. All possible permutations of the retinotopic and non-retinotopic connectivity were then generated by randomly assigning each connectivity strength value to belong to a retinotopic or non-retinotopic connection. Interchanging the retinotopic and non-retinotopic connectivity was restricted to only within the same hemisphere (i.e., retinotopic connectivity of the first hemisphere was not interchanged with non-retinotopic connectivity of the fifth hemisphere and so on) to ensure that total connectivity was preserved (64 permutations, including the original detected total retinotopic and non-retinotopic connectivity). One-tailed *t*-tests over the permuted total connectivity strengths then yielded a *t* value for each permutation. The statistical significance of the detected V1–V2 connectivity patterns was tested at a significance level described by *P* < 0.05. The *t* value obtained for the six original total retinotopic and total non-retinotopic connectivity strengths was then compared with the empirical distribution of *t* values obtained from the permutations, and a permutation *P*-value was derived by taking the proportion of randomly generated *t* values that exceeded or were equal to the observed *t* value for the original connectivity strengths. Separate significance was reported for connectivity patterns obtained for datasets acquired in each of the three sessions at *P* < 0.05.

The reproducibility of V1–V2 connectivity mapping was determined between acquisitions with the 32-channel and flexible surface coils by calculating the intraclass correlation coefficient (ICC) index (Bartko 1966) and coefficient of variation (CoV), first between the six connectivity matrices obtained for each of the two acquisitions with the 32-channel coil and then between the mean of the six connectivity matrices acquired with the two acquisitions using the 32-channel coil and the flexible surface coil. The ICC and CoV matrices were computed using the experimental V1–V2 percentage connectivity matrices for each scan across six hemispheres (of the smaller group scanned in Experiment 1). The ICC and CoV were reported in each case. To further demonstrate reproducibility, the geometry of the mapped V1–V2 fiber tracks was qualitatively investigated.

For the larger group scanned in Experiment 2, *t*he statistical significance of the detected V1–V2 connectivity patterns and the statistical significance of using either the 32-channel or the flexible surface coils were assessed on all corresponding hemispheres common to both 32-channel and flexible surface coil scans for the valid DWI (i.e., those not excluded because of displaced fat signal artifacts). A factorial two-way repeated measures analysis of variance (ANOVA) test was performed with two independent factors “type of connections” (retinotopic and non-retinotopic) and coil (32-channel coil and flexible surface coil). Here, absolute streamline counts were used instead of percentage connectivity in order to render the retinotopic and non-retinotopic connectivity factors independent (i.e., total hemispheric percentage connectivity would always add up to 100%). For each hemisphere, total retinotopic streamline counts detected between all retinotopically corresponding V1 and V2 segments and total non-retinotopic streamline counts corresponding to all other streamlines detected between V1 and V2 segments were used as inputs to ANOVA. Significance was reported for the ANOVA at *P* < 0.05.

## Results

We first present the results of the proof-of-concept study (Experiment 1) on the group of three participants with a detailed qualitative and quantitative analysis. Then, the results of the replication study (Experiment 2) on the larger group of 14 participants are presented to demonstrate reproducibility across fully independent experiments.

### Experiment 1: Proof-of-Concept Study

#### Quality Control: Image SNR, Fiber Estimation, and Tractography

High-quality submillimeter resolution DWI was acquired with both the 32-channel and flexible surface coils for the three participants. The tSNR offered by the flexible surface coil was approximately 1.7 times higher on average compared with the 32-channel coil in superficial brain areas, but less than the 32-channel coil in deep brain areas (ca. 5–7 cm away from the coil surface; Supplementary Fig. 1*b*).

**Figure 2:**
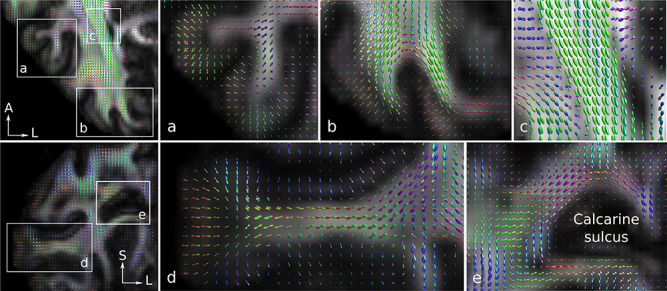
Robust fODF estimates were enabled using submillimeter resolution DWI and MSMT-CSD. The flexible surface coil fODF estimates are shown for a representative participant and are superimposed on the DWI-derived fractional anisotropy map. A, S, and L: anterior, superior, and left, respectively. In a selected axial slice, (*a*) intracortical radial fibers running perpendicular to the cortical GM boundary in the gyri and along the walls of the sulci were detected (see Supplementary Video 1). (*b*) U-fibers running parallel to the cortical GM boundary are shown in a sulcus in the early visual cortex. U-fibers form crossing regions with long-range fibers (Reveley et al. 2015) and obscure their penetration into the cortical GM using submillimeter spatial resolution DWI techniques (see Supplementary Video 2). (*c*) Optic radiation and the posterior tail of the splenium of the corpus callosum tracts were detected to run predominantly anterior–posterior with few or no secondary peaks as expected (see Supplementary Video 1). In a selected coronal slice, (*d*) intracortical radial fibers running perpendicular to the cortical GM boundary in the gyrus and along the wall of the sulcus were reconstructed (see Supplementary Video 3). (*e*) Fibers near the calcarine sulcus were detected to run predominantly parallel to the cortical GM boundary (see Supplementary Video 4).

**Figure 3:**
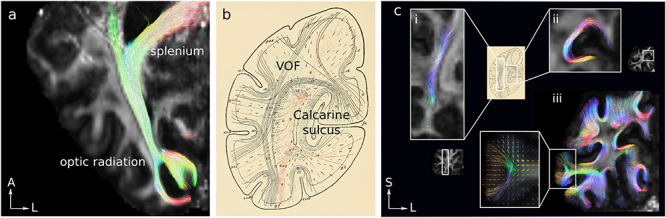
Delineation of known fiber pathways using submillimeter resolution MSMT-CSD probabilistic tractography demonstrated for the flexible surface coil DWI of one representative participant, superimposed on the DWI-derived fractional anisotropy map. (*a*) Fiber tracks corresponding to the optic radiation and the posterior tail of the splenium of the corpus callosum tracts. (*b*) The short fiber connections of the occipital lobe were mapped using histology by Sachs (reproduced from Sachs 1892). Fiber tracks corresponding to (*c-i*) the VOF and (*c-ii*) fibers connecting the upper and lower banks of the calcarine sulcus. (*c-iii*) The short U-shaped fiber tracks mapped by user-defined streamline length and curvature thresholds (see Supplementary Video 5 for a map of the short connections obtained from a 32-channel coil DWI acquisition). U-shaped streamlines penetrated the cortical GM at the gyri. U-shaped streamlines connecting directly adjacent gyral GM are shown in the inset of c-iii. The underlying fODF distribution shows intracortical radial fibers. A, S, and L: anterior, superior, and left, respectively.

The dedicated sequences and hardware setups enabled robust estimation of fODFs with MSMT-CSD even at the submillimeter resolution (see Fig. 2 for a representative DWI acquisition with the flexible surface coil). Detection of intracortical radial fibers was possible, particularly in the gyri close to the coil surface (Fig. 2*a*,*d*). Fibers running predominantly parallel to and directly below the cortical GM–WM boundary corresponded to the U-fibers in the SWM. These dense fibers obscured ascending fibers that penetrated the cortical GM (Fig. 2*b*), in line with a previous ex vivo DWI tractography study (Reveley et al. 2015). Fiber estimates in the optic radiation and the posterior tail of the splenium of the corpus callosum tracts were in line with anatomical expectations and were predominantly anterior–posterior oriented with few or no secondary fiber populations (Fig. 2*c*). Fiber estimates around the calcarine sulcus followed the sulcal folding pattern and were predominantly parallel to the cortical GM boundary in the SWM (Fig. 2*e*). Tractography delineated the optic radiation and the posterior tail of the splenium of the corpus callosum tracts well. The tracts were delineated from the whole-brain tractogram using the retinotopically defined V1 segments and a second user-defined ROI positioned in an approximate anatomical location in the white matter corresponding to the optic radiation tract (Fig. 3*a*).

The short fiber connections in the occipital lobe were delineated from the whole-brain tractogram and qualitatively compared with a nineteenth-century map of U-fibers derived from histology by Sachs (Sachs 1892) (Fig. 3*b*,*c*). Sachs’ detailed map of the short fiber connections in the human occipital lobe featured the vertical occipital fasciculus (VOF) (Yeatman et al. 2014), the short white matter fibers connecting the upper and lower banks of the calcarine sulcus, as well as the short U-shaped white matter connections (Fig. 3*b*). Fiber tracks corresponding to the VOF and the short fibers connecting the upper and lower banks of the calcarine sulcus were delineated manually by user-defined ROIs (Fig. 3,*c-ic-ii*, respectively). The U-shaped connections were also delineated using streamline length and curvature thresholds of 25 mm and 90°, respectively, and are shown on a coronal slice (Fig. 3*c-iii*). Overall, the tractography results were qualitatively similar to Sachs’ early map of the short fiber connections in the occipital lobe and identified many of the short U-shaped connections featured in his work.

#### V1 and V2 Fiber Geometry Mapping

**Figure 4:**
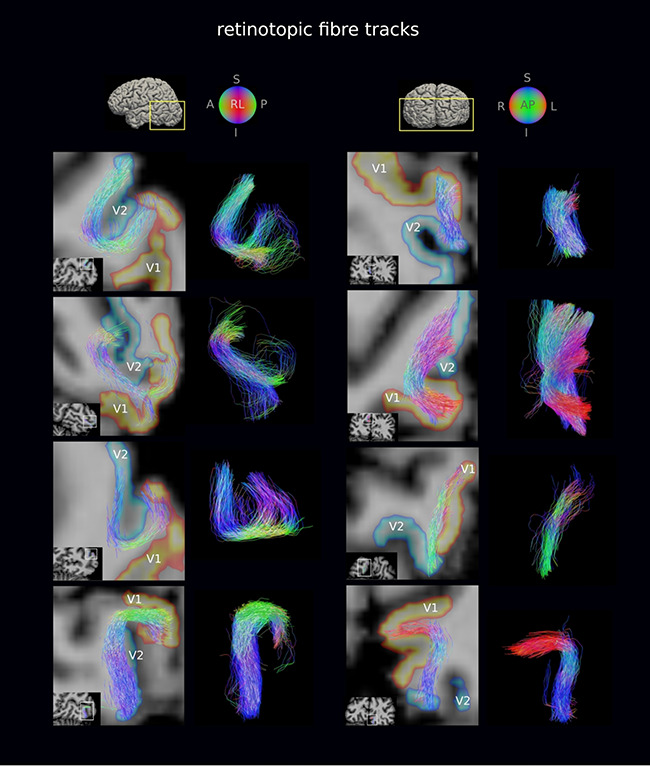
Retinotopic V1–V2 fiber tracks were generally short (16 mm mean length) and followed the pattern of cortical folding. A representative subset of retinotopic V1–V2 fiber tracks are shown for the flexible surface coil DWI of a representative participant. Fiber tracks are shown on oblique slices and are superimposed on the *T*_1_w image transformed to DWI space. The volumetric V1 and V2 retinotopic segments transformed to the DWI space are also shown. The detected retinotopic fiber tracks followed the pattern of cortical folding, but not all were strictly U-shaped. A, L, R, I, S, P: anterior, left, right, inferior, superior, posterior.

**Figure 5:**
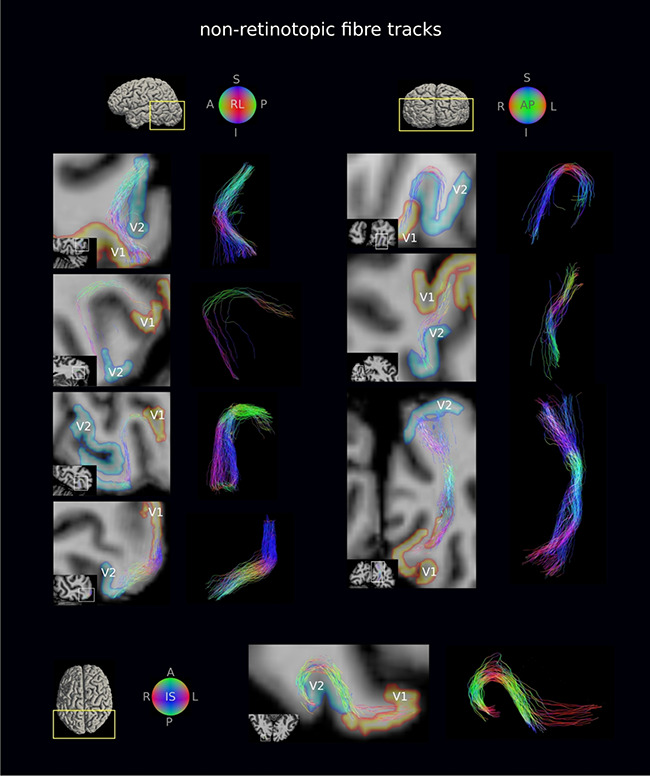
Non-retinotopic V1–V2 fiber tracks were less abundant and on average longer (32 mm mean length) than retinotopic fiber tracks (cf. Fig. 4). A representative subset of non-retinotopic V1–V2 fiber tracks are shown for the flexible surface coil DWI of a representative participant. Fiber tracks are shown on oblique slices and are superimposed on the *T*_1_w image transformed to DWI space. The volumetric V1 and V2 retinotopic segments transformed to the DWI space are also shown. The detected non-retinotopic fiber tracks followed the pattern of cortical folding in many cases, but not all were strictly U-shaped. A, L, R, I, S, P: anterior, left, right, inferior, superior, posterior.

Retinotopic and non-retinotopic V1–V2 fiber tracks were mapped between all pairs of retinotopically defined V1 and V2 segments for each hemisphere. Examples of V1–V2 retinotopic and non-retinotopic fiber tracks are shown on oblique slices for the flexible surface coil DWI acquisition of one representative participant (Figs 4 and 5, respectively). The detected retinotopic and non-retinotopic fiber tracks appeared to run in bundles and followed the pattern of cortical folding closely, as expected for the shorter streamlines in particular, but did not conform to strict U shapes for all fiber tracks.

#### V1 and V2 Connectivity Mapping

**Figure 6:**
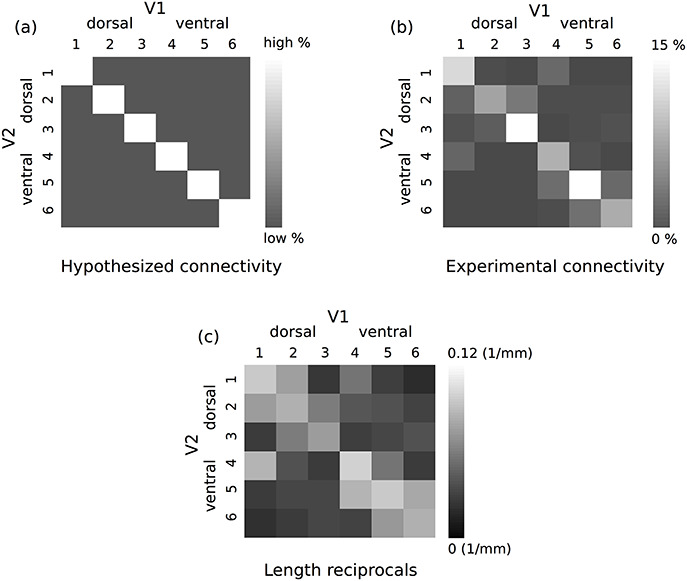
Consistent connectivity patterns were observed between V1 and V2 (see Fig. 1 for the definition of retinotopic segments). (*a*) Hypothesized connectivity matrix with higher expected retinotopic V1–V2 connectivity. The retinotopic and non-retinotopic connections are shown with on- and off-diagonal elements of the connectivity matrix, respectively. (*b*) Group-average V1–V2 connectivity matrix computed across six hemispheres (i.e., three subjects). On average higher connectivity was detected for the V1 and V2 retinotopic segments. (*c*) Corresponding group-average proximity matrix computed based on the reciprocals of the streamline lengths across all subjects and all acquisitions. Retinotopic connectivity showed, on average, higher proximity.

The group-average connectivity matrix of connections between the retinotopically defined V1 and V2 segments created across six hemispheres (i.e., three subjects, two hemispheres each) is shown in Figure 6*b* (see Supplementary Fig. 3 for individual connectivity matrices). As expected, the connectivity between the retinotopically corresponding V1 and V2 segments (Fig. 6*b*, diagonal matrix elements) was on average higher compared with the connectivity between the non-retinotopic segments (Fig. 6*b*, off-diagonal matrix elements). The average contributions of the retinotopic connectivity were significantly higher than of the non-retinotopic connectivity to the total hemispheric V1–V2 connectivity with 72.7% over 27.3% (*P* < 0.05).

The group-average reciprocal length (proximity) matrix estimated for the fiber tracks connecting each retinotopically defined V1 and V2 segment across all subjects and acquisitions is shown in Figure 6*c* (see Supplementary Fig. 4 for per hemisphere reciprocal length matrices). The detected retinotopic connections had on average higher proximity compared with the non-retinotopic connections. The estimated lengths (mean ± standard deviation) of the retinotopic and non-retinotopic fiber tracks across the six hemispheres were 16 ± 4 and 32 ± 13 mm, respectively.

#### Reproducibility of V1 and V2 Connectivity Mapping

**Figure 7:**
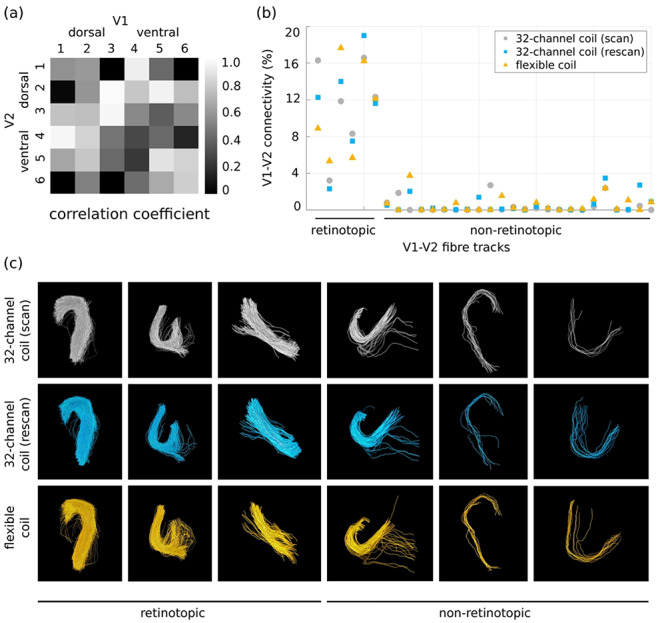
The mapped V1–V2 connectivity is reproducible across scans (scan–rescan) with the 32-channel and the flexible surface coils. (*a*) ICC matrix computed between the mean of the scan–rescan 32-channel coil connectivity and the flexible surface coil connectivity across six hemispheres shows mostly high correlation. (*b*) Percentage connectivity for all detected retinotopic and non-retinotopic fiber tracks using the 32-channel and flexible surface coils averaged over all subjects supports high reproducibility for the three experiments. (*c*) Corresponding fiber track geometries show high reproducibility across the scan–rescan and the flexible surface coil experiments as demonstrated for a representative subset of V1–V2 fiber tracks.

The intraclass correlation coefficient (mean ± standard deviation) was 0.59 ± 0.33 (0.83 ± 0.14 for the retinotopic and 0.55 ± 0.33 for the non-retinotopic connections) (Fig. 7*a*) between the mean of the two 32-channel coil connectivity matrices and the flexible surface coil connectivity matrices and 0.73 ± 0.33 (0.88 ± 0.70 for the retinotopic and 0.69 ± 0.35 for the non-retinotopic connections) between connectivity matrices for the two 32-channel coil acquisitions. The coefficient of variation was 0.23 on average (0.23 ± 0.10 for retinotopic and 0.25 ± 0.14 for non-retinotopic connections). The reproducibility of percentage connectivity obtained for datasets acquired with the 32-channel and flexible surface coils was also demonstrated (see Fig. 7*b* for a representative participant). The geometry of the detected fiber tracks showed high agreement between the 32-channel and flexible surface coil experiments (Fig. 7*c*).

### Experiment 2: Independent Replication Study

#### Quality Control

High-quality submillimeter resolution DWI was acquired with the 32-channel coil for all 14 participants. High-quality DWI was also acquired with the flexible surface coil, however, for only 13 out of the 14 participants. DWI from one participant was severely affected by fat displacement artifacts that could not be avoided during acquisition and was subsequently excluded from the study. Also, significantly higher (*P* < 0.05) motion levels were estimated in the left–right (*P* = 0.0356, *t* = 2.4) and anterior–posterior (*P* = 0.0002, *t* = 5.4) directions in DWI acquired with the 32-channel coil compared with the flexible surface coil.

#### V1 and V2 Connectivity Mapping

**Figure 8:**
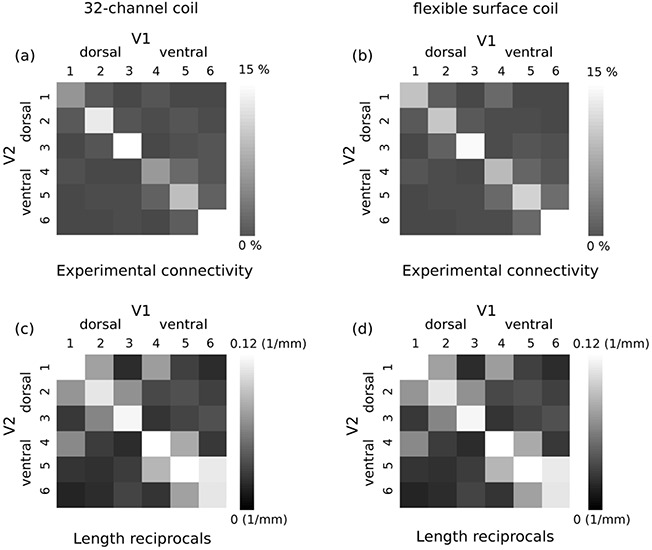
Consistent connectivity patterns between V1 and V2 were reproduced in Experiment 2 on the larger group of 14 participants (see Fig. 1 for the definition of retinotopic segments and Fig. 6*a* for hypothesized connectivity pattern and definition of retinotopic and non-retinotopic connections in the connectivity matrix). (a) Group-average V1–V2 connectivity matrix computed across 28 hemispheres (i.e., 14 subjects) for the 32-channel coil scans. (b) Group-average V1–V2 connectivity matrix computed across 26 hemispheres (i.e., 13 subjects) for the flexible surface coil scans. On average higher connectivity was detected for the V1 and V2 retinotopic segments for both 32-channel and flexible surface coil DWI. (c,d) Corresponding group-average proximity matrices computed based on the reciprocals of the streamline lengths across all hemispheres. Retinotopic connectivity showed, on average, higher proximity.

The group-average connectivity matrices of connections between the retinotopically defined V1 and V2 segments created across 28 hemispheres (i.e., 14 scans, 2 hemispheres each) for the 32-channel coil and 26 hemispheres (i.e., 13 scans, 2 hemispheres each) for the flexible surface coil are shown separately in Figure 8*a*,*b*. Similar to the results of Experiment 1, connectivity detected between the retinotopically corresponding V1 and V2 segments (Fig. 8a,*b* diagonal matrix elements) was on average higher compared with the connectivity between the non-retinotopic segments (Fig. 8*a*,*b*, off-diagonal matrix elements). The average contributions of retinotopic and non-retinotopic connectivity to the total hemispheric V1–V2 connectivity were 78.3% and 21.7% for the 32-channel coil and 72.8% and 27.2% for the flexible surface coil. The retinotopic connectivity was significantly higher than non-retinotopic, as shown by the two-way ANOVA (*P* < 0.05).

The group-average reciprocal length (proximity) matrix estimated for the fiber tracks connecting each retinotopically defined V1 and V2 segment across all hemispheres is shown in Figure 8*c*,*d* for the 32-channel and flexible surface coils, separately. The detected retinotopic connections had on average higher proximity compared with the non-retinotopic connections. The estimated lengths (mean ± standard deviation) of the detected retinotopic and non-retinotopic fiber tracks were on average 11 ± 4 and 26 ± 16 mm for the 32-channel coil scans (i.e., 28 hemispheres) and 12 ± 3 and 26 ± 14 mm for the flexible surface coil scans (i.e., 26 hemispheres), respectively.

The two-way ANOVA test revealed a main effect of type of connections with significantly higher number of retinotopic in comparison with non-retinotopic streamlines (significance level of *P* < 0.05, *F* = 99.8, *P* = 1.1 × 10^−16^). No effect of coil (*F* = 0.54, *P* = 0.46) and no interaction between type of connections and coil were revealed by the test (*F* = 0.49, *P* = 0.48).

## Discussion

In this study the short association fibers (U-fibers) in the V1 and V2 processing stream were mapped in the human brain noninvasively in vivo using a combination of submillimeter resolution DWI-based probabilistic tractography and fMRI-based retinotopy. This unique combination of functional and structural imaging has shown—to the best of our knowledge for the first time in the human brain in vivo—patterns of short white matter connectivity between V1 and V2, which follow the retinotopic organization principle. Specifically, the detected V1–V2 connectivity (relative streamline counts) was found to preferentially connect retinotopically corresponding areas in V1 and V2 (72.7% contribution to total V1–V2 connectivity vs. 27.3%), in line with the principle of retinotopic organization in the primate brain (Holmes 1918, 1945; Tootell et al. 1988; Felleman and Van Essen 1991) as demonstrated in a small group proof-of-concept experiment. The results of the initial experiment were corroborated by a second independent study in a larger group of 14 participants, which found the same retinotopic connection pattern.

The geometry of the V1–V2 fiber tracks followed the pattern of cortical folding, as expected for the shorter streamlines in particular, but did not conform to strict U shapes for all fiber tracks (Figs 4 and 5). In light of this finding, the use of length and curvature filters for U-fiber mapping (e.g., Zhang et al. 2014; O’Halloran et al. 2017; De Santiago et al. 2017a, 2017b; Li et al. 2019) should be considered carefully. The detected V1–V2 connectivity was reproduced across repeat scans using the 32-channel coil, for setups using the flexible surface coil and in a separate independent experiment. Taken together, our presented submillimeter DWI tractography approach using the 3-T Connectom MRI scanner proved feasible and reliable for mapping the short association fibers in the human brain.

### A Three-Component Approach to U-Fiber Mapping

Our presented approach has important implications which we now discuss. Techniques for mapping the short association fibers (U-fibers) are essential for both basic neuroscientific and clinical investigations because of the important role these fibers play in brain development, aging, and function as well as being implicated in pathology. However, the connectivity facilitated by the short association fibers is largely unexplored in humans. The development of validated in vivo DWI tractography procedures for U-fiber connectivity mapping is an essential step toward understanding their roles in the brain as well as contributing to a more complete human brain connectome. Importantly, DWI in the SWM is particularly challenging because the region occupied by the fibers is narrow, forms regions of crossing afferent and efferent cortical fibers, and is affected by partial voluming with the overlying cortical gray matter. We presented a sensitive and robust method to address the challenges of U-fiber mapping using in vivo DWI. Our approach consists of three key components: 1) high-quality submillimeter DWI acquisition utilizing cutting-edge high-performance gradients; 2) dedicated preprocessing, fiber modeling, and tractography; and 3) multimodal in vivo partial validation performed in the same individual.

High-quality submillimeter DWI acquisition, essential for robust tractography of short association fibers (Setsompop et al. 2013; Song et al. 2014), was facilitated using the high-performance gradients implemented in the 3-T Connectom scanner. This allowed us to reduce the echo time from approximately 75 ms achievable on high-performance MRI scanners also used for clinical applications (e.g., 3-T Prisma, Siemens Healthineers) to 64 ms used in this study, corresponding to an increase in SNR of approximately 18% assuming the transverse relaxation time (*T*_2_) of approximately 65 ms in the white matter. Compensating for the lower SNR would require an increase of approximately 40% in measurement time, which may lead to compliance issues and exceed maximal scan session durations.

Further, we tested whether a high-sensitivity flexible surface coil that yielded high signal sensitivity proximal to the surface of the coil would improve U-fiber mapping efficiency in the posterior occipital lobe. Interestingly, the flexible surface coil provided very similar U-fiber mapping results compared with the 32-channel coil (Figs 7 and 8; Supplementary Fig. 3), despite its high sensitivity in superficial brain areas (Supplementary Fig. 1). This might be explained by tractography performance being influenced more by the sulcal regions further away from the coil, where the sensitivities of the two coils were comparable (Supplementary Fig. 1).

The 32-channel and flexible surface coils also differed in how their DWI was affected by artifacts, for example, from displaced fat signal from the scalp and motion. The high sensitivity of the flexible surface coil to nearby structures also increases the fat signal from the scalp, which can be shifted to inside the brain. This problem can be partly overcome by adjusting the imaging FOV to shift it away from the regions of interest, but it cannot resolve the problem for all subjects owing to differences in brain size and geometry. Moreover, the estimated translational motion of the flexible surface coil DWI was lower than that estimated for the 32-channel coil and may be explained by the tight padding used for imaging with the flexible surface coil.

The dedicated analysis procedure included careful preprocessing consisting of correction for gradient nonlinearities and susceptibility- and eddy current-related artifacts, significantly improving the spatial specificity of the DWI. Crossing fibers and partial volume effects in SWM were addressed by employing a model that accounted for both effects (Dhollander et al. 2016; Theaud et al. 2018), and fiber pathways were reconstructed using a tractography method designed to track robustly through regions of high curvature (Tournier et al. 2010).

Multimodal partial validation was the third key component of our approach. Although the high reproducibility of the mapped connectivity supports the validity of the presented approach, it remains a challenge to fully validate it (and indeed tractography in general). Validation of tractography is inherently difficult because ground-truth information is difficult to obtain (Fillard et al. 2011; Côté et al. 2013; Hubbard and Parker 2013; Knösche et al. 2015; Sotiropoulos and Andrew 2017; Jeurissen et al. 2019; Schilling et al. 2019). It is further complicated for U-fiber connectivity mapping because of the limited available knowledge of their geometries, trajectories, and distributions. To address these challenges, we capitalized on the known structure–function relationship in the visual processing stream, which predicts a preference for retinotopic connections between different areas in V1 and V2. The converging results for the expected functional connectivity pattern between the retinotopic segments in V1 and V2 measured with functional MRI retinotopy and the DWI-based structural connectivity results provide further support for the validity of the presented approach. While acknowledging that comprehensive validation strictly requires direct comparison against a ground-truth reference, we believe that the direct comparison of functional neuroanatomy—here retinotopy—and structure is a very useful part of and contributes strong evidence for a multifaceted validation approach. Moreover, another important contribution to validation is the demonstration of reproducibility. This in vivo approach has the advantage that it can be performed in the same individual and avoids tissue changes in postmortem validation experiments, which can significantly affect the results (Edwards et al. 2018).

### Considerations and Limitations

Our findings may have been affected by a number of confounding factors originating from diffusion tractography and functional retinotopy.

Tractography has inherent limitations that can lead to biases and errors in fiber mapping, even when based on high-quality diffusion MRI data (Thomas et al. 2014; Knösche et al. 2015; Sotiropoulos and Andrew 2017). A bias of streamline tractography toward detection of short streamlines has been previously demonstrated (Jones 2010; Jbabdi and Johansen-Berg 2011; Ercsey-Ravasz et al. 2013; Donahue et al. 2016; Schilling et al. 2018; Jeurissen et al. 2019). This length bias might have affected our assessment of the retinotopic connectivity principle because of differences in the average estimated lengths of the V1–V2 retinotopic and non-retinotopic connections (11 ± 4 and 26 ± 16 mm, respectively, in Experiment 2). However, the observed difference in the lengths may also be at least partially biologically driven, for example, due to the suggested biological inverse relationship between fiber length and abundance in the primate brain (Schüz and Braitenberg 2002; Markov et al. 2013) (see also Supplementary Fig. 5). Complex fiber arrangements near the SWM also pose a challenge for tractography (Supplementary Fig. 6*a*). Moreover, almost all detected fiber tracks terminated in the gyral crowns (Figs 4 and 5; Supplementary Fig. 6*b*) in line with the well-known gyral bias effect (Van Essen et al. 2013; Schilling et al. 2018). In contrast to this observation, the retinotopic projection principle implies that all receptive fields within V1 project to all retinotopically corresponding receptive fields within V2, that is, full coverage of the cortical surface by fiber tracks.

V1 and V2 retinotopic segmentation errors may have additionally affected our assessment of the retinotopic connectivity principle via misclassification of retinotopic fiber tracks into non-retinotopic ones and vice versa (Supplementary Fig. 6*c*). Particularly, registration inaccuracies from functional MRI to DWI space may have introduced further issues such as gaps and overlaps between the mapped cortical areas (Supplementary Fig. 6*c*). Since the average relative size of the gaps and overlaps between retinotopic segments was below 11% of their total areas, we are confident that the achieved precision was sufficient to justify the main result of the study (see Supplementary Fig. 2).

Although we believe that the in vivo assessment of the convergence of structural connectivity measured by diffusion tractography and functional connectivity predicted by functional retinotopy is a powerful and critical test, we note that it relies on a strong prediction based on previous work in primates (Felleman and Van Essen 1991). Therefore, we are currently conducting further experiments, which compare postmortem MRI to advanced histological methods (e.g., CLARITY [Morawski et al. 2017]) applied in human tissue, in order to provide independent support.

Importantly, we restricted the scope of the present work to mapping only the short white matter connections between V1 and V2. Lateral connectivity within V1 and V2 realized mostly by intracortical fibers in primate early visual processing stream (Barone et al. 2000; Angelucci et al. 2002; Stettler et al. 2002) was not assessed. Current in vivo DWI tractography does not support connectivity mapping within the cortex, and only a few ex vivo DWI tractography studies demonstrated intracortical fiber mapping (Leuze et al. 2014; Aggarwal et al. 2015). However, our approach could be extended to mapping the short white matter V2–V3 and V1–V3 connections, which are known to be retinotopically organized.

### Outlook

In future studies, our approach could be extended to map the connectivity in higher visual cortical areas where the retinotopic projection principle also holds or in other networks of connections, for example, the language processing networks, where well-defined hypotheses are laid out (Friederici 2011). Advanced diffusion MRI techniques such as multiband (Moeller et al. 2010; Setsompop et al. 2012b; Xu et al. 2013) or gSlider (Setsompop et al. 2018) could be used to improve acquisition SNR and enable even higher-resolution or greater FOV coverage. Alternative methods of V1 and V2 segmentation could be used based solely on anatomical landmarks (Benson et al. 2012, 2014) or Bayesian methods (Benson and Winawer 2018) to minimize the acquisition time. Advanced tractography techniques (e.g., Smith et al. 2013, 2015a, 2015b; Daducci et al. 2015, 2016; Girard et al. 2014, 2017; St-Onge et al. 2018) and dedicated tracking strategies (Schilling et al. 2018) may be used to improve tractography in the SWM. Future advances in methods may also make it possible to assess connectivity within the cortex itself (currently only demonstrated in a few studies in the postmortem brain using diffusion MRI tractography [Leuze et al. 2014; Aggarwal et al. 2015]), which would complement U-fiber and long-range fiber mapping and contribute to complete characterization of connectivity in the human brain.

## Conclusions

The retinotopically organized short association fibers (U-fibers) connecting V1 and V2 cortical areas were mapped in the human brain noninvasively in vivo. Our framework provides a major step toward understanding the roles of short association fibers in brain development, function, and information processing as well as involvement in pathology and contributes to the construction of a more complete human brain connectome.

## Funding

The research leading to these results has received funding from the European Research Council under the European Union’s Seventh Framework Programme (FP7/2007-2013)/ERC grant agreement n° 616905. NW received funding from the BMBF (01EW1711A & B) in the framework of ERA-NET NEURON, and from the NISCI project funded by the European Union’s Horizon 2020 research and innovation programme under the grant agreement No 681094, and the Swiss State Secretariat for Education, Research and Innovation (SERI) under contract number 15.0137.

## Notes

We thank the University of Minnesota Center for Magnetic Resonance Research for the provision of the multiband EPI sequence software. We especially thank Dr Saskia Helbling for insightful advice, Domenica Wilfling for supporting the data acquisition, Dr Thomas Witzel for providing the MGH diffusion sequence, and Dr Martin Sereno for providing support with the retinotopy experiment. We also thank Dr Alfred Anwander for helpful discussions and the International Max Planck Research School on Neuroscience of Communication: Function, Structure, and Plasticity for their support. *Conflict of Interest:* The Max Planck Institute for Human Cognitive and Brain Sciences has an institutional research agreement with Siemens Healthineers. N.W. was a speaker at an event organized by Siemens Healthineers and was reimbursed for the travel expenses.

## Supplementary Material

suppl_data_bhaa049
