# Supplementary material for: Mapping Short Association Fibers in the Early Cortical Visual Processing Stream Using In Vivo Diffusion Tractography
Source: Cereb Cortex. 2020 Apr 8;30(8):4496–514. doi: 10.1093/cercor/bhaa049 (PMC7325803; doi:10.1093/cercor/bhaa049)
Supplement: suppl_data_bhaa049 [file suppl_data_bhaa049.zip › suppl_data_bhaa049/Supplementary Figure Legends.docx]

Figure S1. Flexible surface RF receive coil provides higher tSNR in superficial brain regions compared to 32-channel RF receive coil. (a) The flexible surface coil is a 23-channel phased array coil designed for high quality imaging of the occipital cortex (Fras-Kriegl et al. 2018). The flexibility of the coil allows for adapting its curvature to the individual head shape, to achieve a tight fit, ensure minimum distance to the coil surface and achieve high SNR. A wooden frame was designed to optimize the fit and mechanical stability. In this study the strap was not wrapped around the participants’ heads as shown in the image, to ensure comfort. (b) Time-series (b=0 s/mm2) tSNR maps are shown on sagittal and axial slices for both RF coils for a representative participant. The flexible surface coil has higher tSNR close to the coil surface in superficial brain areas, but it rapidly drops off with distance from the coil surface. The 32-channel coil has lower tSNR in superficial brain areas but provides a more uniform spatial distribution. Two regions of interest (ROI), proximal and distal to the coil surface, were defined in superficial and deep brain areas for tSNR quantification. (c) Mean tSNR for the flexible surface coil was approximately 1.7 times higher compared to mean tSNR for the 32-channel coil in the superficial ROI, but dropped below that of the 32-channel coil in the deep ROI. For the flexible surface coil, the tSNR was almost four times higher in superficial (11.12 ± 5.12) compared to deep (3.35 ± 0.96) brain areas, whereas for the 32-channel coil, the tSNR was approximately 1.4 times higher in superficial (6.75 ± 2.7) compared to deep (4.72 ± 1.23) brain areas.

Figure S2. Retinotopically defined V1 and V2 segments presented on the inflated cortical surface (top) and volumetric DWI-derived FA map (bottom), both for the right hemisphere of one representative participant. (a) The field sign map was computed and shows borders between V1 and V2. (b) V1 and V2 were manually segmented on the surface, following the V1 and V2 borders carefully to avoid overlapping areas. (c) V1 and V2 were further delineated into six retinotopic segments, corresponding to the six visual hemifield projections on the cortical surface. The outlines of the resulting segments were manually refined. Care was taken to avoid overlaps between the final V1 and V2 segments. (d-f) Labelled V1 and V2 retinotopic segments transformed to volumetric DWI space in axial, coronal and sagittal slices, respectively. The V1 and V2 segmentation and interpolation steps created overlapping and non-contiguous segments that affected ca. 2.2% and 11% of the total V1 and V2 volume area (averaged across all six hemispheres in the proof-of-concept Experiment 1), respectively. A, P, L, R, I, S: anterior, posterior, left, right, inferior, superior. Figure S3. (a-f) V1–V2 connectivity matrices show high reproducibility across scan–rescan with 32-channel coil and flexible surface coils for six hemispheres in the proof-of-concept Experiment 1 (see Fig. 1 for definition of retinotopic segments).

Figure S3. (a-f) V1–V2 connectivity matrices show high reproducibility across scan–rescan with 32-channel coil and flexible surface coils for six hemispheres in the proof-of-concept Experiment 1 (see Fig. 1 for definition of retinotopic segments).

Figure S4. (a) V1–V2 connectivity matrices averaged across the test-retest experiments (as part of the proof-of-concept Experiment 1) conducted with the 32-channel coil for six hemispheres separately (see Fig. 1 for definition of retinotopic segments). (b) mean of the reciprocal lengths of the corresponding connections.

Figure S5. Differences in average fibre track lengths between retinotopic and non-retinotopic fibres cannot be explained by tractography biases only. The streamline counts obtained from whole-brain tractography followed an exponential-like curve decreasing with streamline length. The 32-channel coil DWI tractography results were pooled across fourteen subjects (replication study in Experiment 2) for whole-brain, retinotopic and non-retinotopic connectivities (the flexible surface coil tractography results were excluded because of the low sensitivity of the coil in deep brain areas). The ratio of retinotopic to non-retinotopic streamline counts was higher (3.50) than ratio of total counts detected (1.50) at corresponding lengths (11 mm and 26 mm for the retinotopic and non-retinotopic average lengths, respectively). Whole-brain tractograms likely reflect both biological causes and tractography bias. The greater ratio of the counts at the two lengths corroborates that tractography performance was not only driven by inherent tractography biases for the observed pattern of retinotopic and non-retinotopic connectivity.

Figure S6. (a) Complex fibre distributions, (b) gyral bias and (c) retinotopic segmentation errors as potential sources of false positive and negative retinotopic/non-retinotopic fibre tracks. (a) Tractography is difficult in regions with complex fibre arrangements. Two instances of long ( > 3 cm) non-retinotopic fibre tracks are shown featuring suspicious deviations in their trajectories. Close inspection of the fODFs (left) shows complex fibre arrangements that challenge tractography algorithms. Fibre tracks detected between V1(5)–V2(2) and V1(3)–V2(6) (middle) may have been part of an adjacent fibre pathway connecting V1(5)–V1(2) and V1(3)–V1(6) (right), respectively but were classified to penetrate V2(2) and V2(6), respectively (false positives). Alternatively, the low fODF resolution in the crossing fibre region may have given rise not only to deviations in trajectories of the fibre tracks but also fewer streamlines detected between V1(5)–V2(2) and V1(3)–V2(6) (false negatives). (b) Point-to-point V1–V2 connectivity mapping was not achieved with in vivo probabilistic tractography. Not all points in V1 were found to connect to their retinotopically corresponding points in V2, creating partial coverage of the cortical surface. This was likely driven by gyral bias, regions with complex fibre distributions and gaps between the retinotopically defined V1 and V2 segments. Gaps covered almost 11% of the total volume of V1 and V2 segments. Depending on whether the gaps appeared at the sulci or gyri, the effect on the detected streamlines connecting V1 and V2 could be different. The geometry of the cortical folding pattern, the U-fibres in the SWM and tractography parameter setup may also contribute to observed gyral bias effects (Van Essen et al. 2013; Shilling et al. 2018). Fibre ODFs are shown in a sulcal regions (i-iv). (i) fODF distribution directly below the sulcus is complex and the reconstructed fODFs are small and likely ignored by tractography. Other fODFs run parallel to the sulcal wall and do not penetrate it. (ii) Fibres running parallel to the GM–WM boundary prevent detection of other fibres penetrating the cortical GM at or along the walls of the sulcus. (iii) Fibres running parallel to the GM–WM boundary connect V1 and V2 at the gyri. (iv) Complex fibre arrangements directly below the sulcus do not show support for tracking into cortical GM at or near the sulcus. (c) Fibre classification is likely affected by the precision of V1 and V2 retinotopic segmentations. Streamlines connect V1(6)-V2(6) and V1(6)-V2(5) at the border between the two V2 segments, classified into retinotopic and non-retinotopic connections, respectively. The geometry of the detected retinotopic and non-retinotopic fibre tracks is very similar. The adjacency of the streamline termination points in the two different V2 segments exemplifies the difficulty in assigning them to retinotopic or non-retinotopic connections.
